# Development and evaluation of a Diet Quality Index for preschool children in an Asian population: the GUSTO cohort

**DOI:** 10.1016/j.jand.2022.06.013

**Published:** 2022-06-18

**Authors:** Maryann Regina Rolands, Jia Ying Toh, Ray Sugianto, Wen Lun Yuan, Yung Seng Lee, Kok Hian Tan, Fabian Yap, Keith M Godfrey, Johan G Eriksson, Yap-Seng Chong, Klazine Van der Horst, Mary Foong-Fong Chong

**Affiliations:** Bern University of Applied Sciences, Länggasse 85, 3052 Zollikofen, Bern Switzerland; Singapore Institute for Clinical Sciences, Agency for Science, Technology and Research, A*STAR, Singapore 117609, Singapore; Saw Swee Hock School of Public Health, National University of Singapore and National University Health System, Singapore, Singapore; Singapore Institute for Clinical Sciences, Agency for Science, Technology and Research (A*STAR); Singapore Université de Paris, CRESS, Inserm, INRAE, F-75004 Paris, France; Singapore Institute for Clinical Sciences, Agency for Science, Technology and Research (A*STAR), Singapore; Department of Pediatrics, Yong Loo Lin School of Medicine, National University of Singapore, Singapore; Division of Paediatric Endocrinology, Khoo Teck Puat-National University Children's Medical Institute, National University Hospital, National University Health System, Singapore; Duke-National University of Singapore Graduate Medical School, Singapore; Department of Maternal Fetal Medicine, KK Women’s and Children’s Hospital, Singapore; Duke-National University of Singapore Graduate Medical School, Singapore; Department of Pediatric Endocrinology, KK Women's and Children's Hospital, Singapore; Lee Kong Chian School of Medicine, Nanyang Technological University, Singapore; Medical Research Council Lifecourse Epidemiology Centre, University of Southampton, Southampton, United Kingdom; National Institute for Health Research Southampton Biomedical Research Centre, University Hospital Southampton, NHS Foundation Trust, Southampton, United Kingdom; Singapore Institute for Clinical Sciences, Agency for Science, Technology and Research (A*STAR), Singapore; Department of Obstetrics & Gynaecology, Yong Loo Lin School of Medicine, National University of Singapore Singapore; Folkhälsan Research Center, Helsinki, Finland; Department of General Practice and Primary Health Care, University of Helsinki and Helsinki University Hospital, Helsinki, Finland; Singapore Institute for Clinical Sciences, Agency for Science,Technology and Research (A*STAR), Singapore; Department of Obstetrics & Gynaecology, Yong Loo Lin School of Medicine, National University of Singapore, Singapore; Department Nutrition and Dietetics, Faculty of Health Professions, Bern University of Applied Sciences, Murtenstrasse 10, 3012 Bern, Switzerland; Singapore Institute for Clinical Sciences, Agency for Science, Technology and Research (A*STAR), Singapore; Saw Swee Hock School of Public Health, National University of Singapore and National University Health System, Singapore

**Keywords:** Diet quality index, Development, Evaluation, pre-school children, Asia, diet quality

## Abstract

**Background:**

Diet quality indices (DQIs) are useful tools to measure diet quality as they compare dietary intakes against recommendations. A DQI for Asian preschool children is lacking.

**Objective:**

The aims of this study were to develop and evaluate a DQI for preschool children based on Singapore dietary recommendations and to examine diet quality in a cohort of 5-year-old children. An additional aim was to assess associations between sociodemographic characteristics and DQI-5 scores.

**Design:**

A secondary analysis was conducted using dietary intake of children from the Growing Up in Singapore Towards healthy Outcomes (GUSTO) mother-offspring cohort assessed in 2015-2016 using a validated Food Frequency Questionnaire. The socio-demographic data was assessed at recruitment between June 2009 and September 2010. The DQI was evaluated using a construct validity approach, whereby nutritional parameters associated with diet quality were studied.

**Participants/setting:**

Participants were 767 Singaporean children aged 5 years of Chinese, Malay or Indian ethnicity.

**Main outcome measures:**

The main outcome measures were the DQI-5 scores and the sociodemographic characteristics associated with diet quality.

**Statistical analyses performed:**

Kruskal-Wallis tests were used to evaluate differences in adherence to dietary recommendations across DQI-5 tertiles. Linear multiple regression analysis was performed to identify sociodemographic characteristics that were associated with diet quality in the children.

**Results:**

The DQI-5 consists of 12 food and nutrient components, with a minimum score of zero and a maximum score of 110 points. The higher scores indicate a healthier diet, the mean (SD) DQI-5 score for the children was 61.6 (13.2). DQI-5 components with low scores included wholegrains, vegetables and fatty acid ratio, while total rice and alternatives, milk and dairy products components were overconsumed by 18% and 24.4% of the children, respectively. Children with higher scores were more likely to meet dietary recommendations and had higher intake of nutrients such as dietary fibre, iron, vitamin A and beta carotene. Children whose mothers were of Malay ethnicity and whose mothers had low income, an education below university and shared primary caregiver responsibilities were more likely to have lower DQI-5 scores.

**Conclusions:**

The DQI-5 scores revealed diets to be low for several components and excessive for a few. The DQI-5 developed for preschool children in Singapore had adequate construct validity.

## Introduction

Promoting healthy eating in early childhood is important to help establish healthy eating habits that can track to later years^1^. There is clear evidence demonstrating that children who adhere more closely to recommendations for diet and lifestyle have lower risks of developing obesity, metabolic syndrome and cardiovascular diseases^2, 3^. Diet quality indices (DQIs) measure how a population’s dietary intake conforms to dietary recommendations^4^. As DQIs are based on national dietary recommendations, they are suitable tools for assessing the healthfulness of local dietary habits^4^. Furthermore, DQIs are useful tools to ascertain how well the local population has adhered to current recommendations^4^. Only few DQIs have been developed and evaluated for Asian paediatric populations^5, 6^. To date in Singapore, there is a DQI developed and evaluated for toddlers ^7^ but none for pre-schoolers.

The literature suggests that there is no gold standard to which a newly developed DQI can be compared with to reference its validity^8^. Instead, the use of construct validity is an approach that has been used to evaluate the DQI ^8^. For example, higher scores derived from an Australian DQI were associated with a “healthy” diet characterised by higher intakes of fruit, vegetables, cereals, vitamin C, folate, and iron, as well as greater diet variety^9^.

The main aims of this study were to develop a DQI for preschool children (DQI-5) using the Singapore dietary recommendations for children 3 to 6 years of age and to evaluate the validity of its construct. Using GUSTO cohort data, the diet quality of Singaporean preschool children aged 5 years was assessed using the DQI-5. Additionally, a secondary aim was to assess associations between sociodemographic characteristics of GUSTO participants with DQI-5 scores to identify population subgroups at risk for low diet quality.

## Methods

### Study population

**Figure 1:**
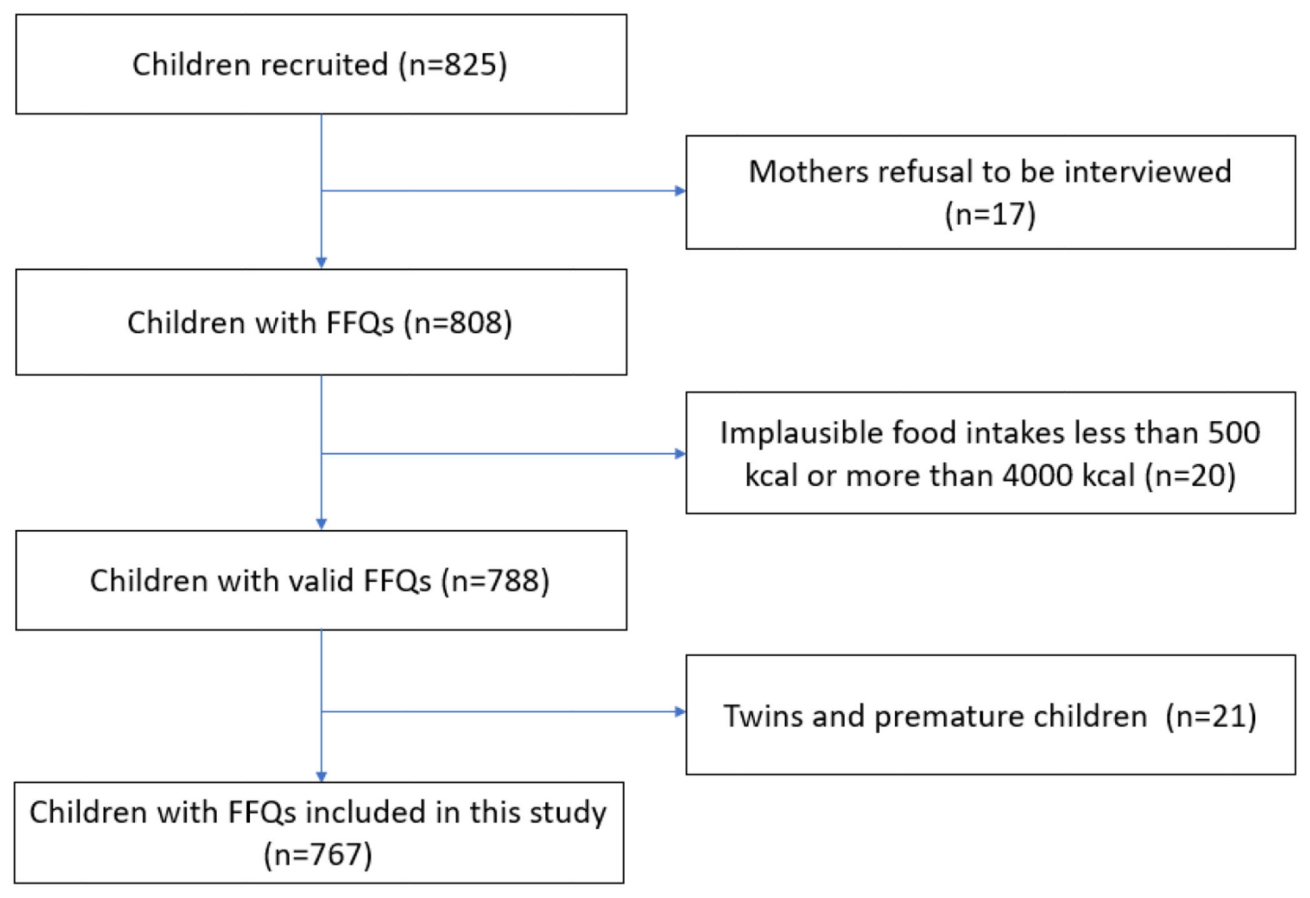
Flowchart of children from the Growing Up in Singapore Towards healthy Outcomes cohort from 2015-2016 included in this study

The data used in this study was obtained from the Growing Up in Singapore Towards healthy Outcomes (GUSTO), a multi-ethnic mother-offspring cohort with a focus on early influences on the development of obesity and metabolic outcomes among children. A detailed cohort design and protocol has been previously published^10^. The study recruited pregnant women of Chinese, Malay, and Indian ethnicities aged 18 to 50 years old, who attended their first trimester antenatal appointments at Singapore’s two main maternity units. The investigators classified the women to be female sex and they were determined to be pregnant by ultrasound scans as part of the antenatal appointment. Pregnant women who were undergoing chemotherapy, on psychotropic drugs or had type 1 diabetes mellitus were excluded. The GUSTO cohort had a total of 1176 babies, with 825 children recruited for this study (see Figure 1). The Institutional Review Board of KK Women’s and Children’s Hospital and National University Hospital approved the study and written informed consent was obtained from the mothers at recruitment (ClinicalTrials.gov Identifier: NCT01174875).

### Development of DQI-5

The DQI-5 was constructed based on the approach taken by Chen et al. (2019) in creating a DQI for 18-month-old toddlers in Singapore^7^, and further adapted to account for greater diet variety in preschool children. The DQI-5 was developed to reflect the Singapore dietary recommendations for three- to six-year-olds and the Singapore Healthy Meals in Childcare Centers Programme guidelines^11-13^. The current dietary recommendations focus on three core food categories: brown rice and wholemeal bread, fruits and vegetables and meat and others^12^. The other dietary recommendations include reducing intake of high sugar food and drinks, consuming healthier oils, and having variety in the diet.

There is no specific recommendation for the maximum servings of high sugar foods for preschool children in Singapore, thus the recommended consumption of added sugar for the general population was followed^14^, which is stipulated as no more than 10% of daily energy intakes (approximately 35 grams daily for pre-schoolers). This is similar to the maximum servings used in the DQI at 18 months in the GUSTO cohort^7^. Additionally, the fatty acid ratio component was adapted from the US Healthy Eating Index-2015^15^ to ascertain the quality of the fats/oils consumed by the children.

**Figure 2:**
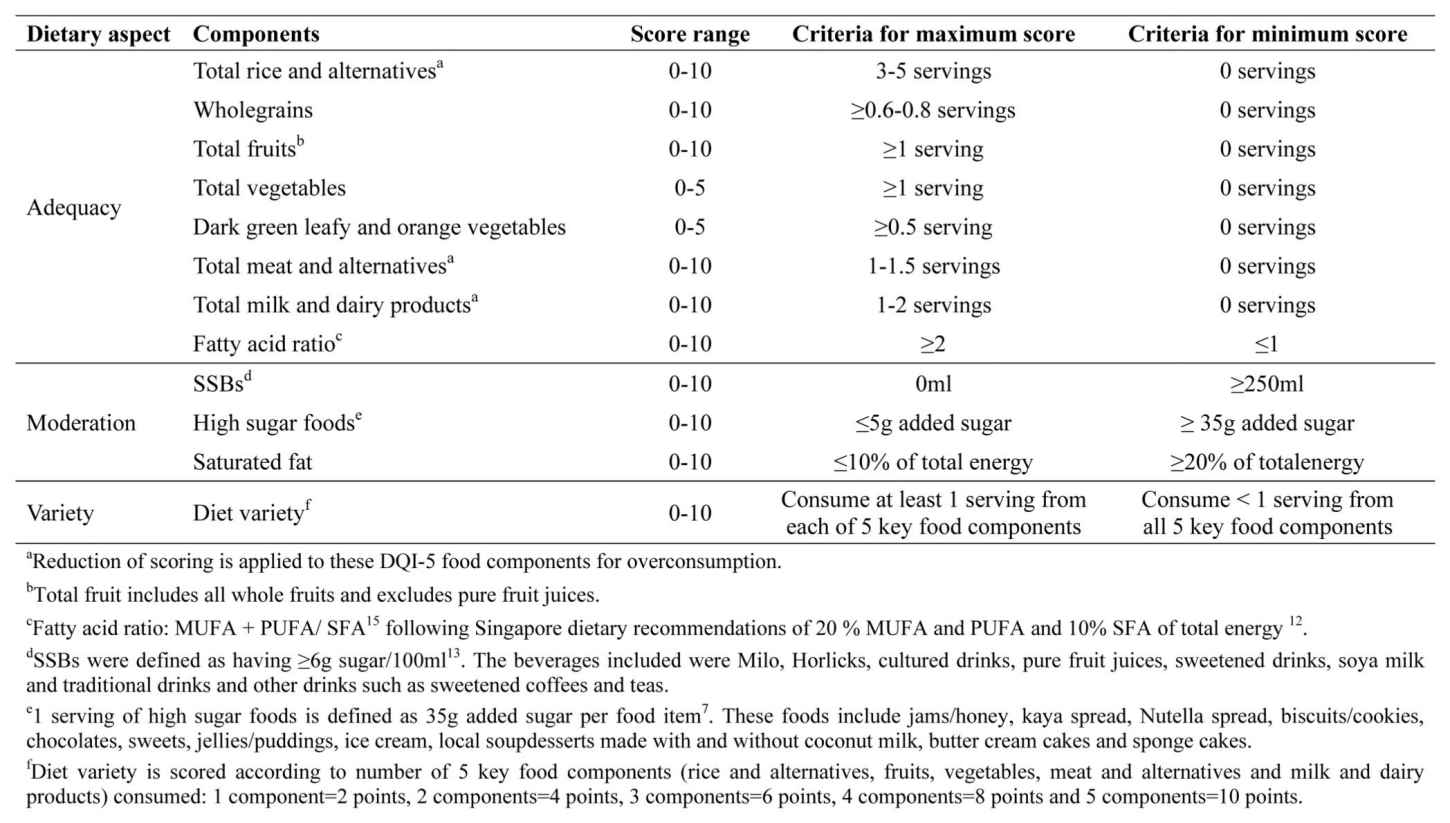
Diet quality index scoring criteria for 5-year-old children in the Growing Up in Singapore Towards healthy Outcomes cohort from 2015-2016.

The DQI-5 is comprised of a total of 12 components to reflect the dimensions of a healthy diet including adequacy, moderation and variety. As presented in Figure 2, eight components represented foods which should be consumed adequately (rice and alternatives, wholegrains, vegetables, dark green leafy and orange vegetables, fruits, meat and alternatives, milk and dairy products and fatty acid ratio), three components represented foods to be consumed in moderation (high sugar foods, sugar sweetened beverages (SSBs), and saturated fat) and the remaining component represented diet variety.

### Weightage and scoring system of DQI-5 components

As shown in Figure 2, each core food category was assigned 20 points, which was further subdivided between diet adequacy and quality^15, 16^. For example, the brown rice and wholemeal bread food category had 10 points allocated for adequacy of total rice and alternatives foods consumed and another 10 points for wholegrain intakes to reflect the quality of grains consumed. Similarly, the meat and others food category had 10 points allocated for adequacy of meats and alternatives consumed (e.g., 2 servings) and 10 points for milk and dairy products to reflect the quality of proteins consumed. The fruits and vegetables food category had 10 points for total fruits consumed and the remaining 10 points divided into five points for adequacy of vegetables consumed and five points for quality of vegetables consumed (i.e., dark green leafy and orange vegetables). The remaining components, which were fatty acid ratio, high sugar foods, SSBs, saturated fats and diet variety, were allocated 10 points each^16^. Overall, the DQI-5 gave a minimum score of zero and a maximum score of 110 points. Higher scores from the DQI-5 indicate a greater adherence to dietary recommendations. Each DQI-5 component was scored based on the extent to which a child met dietary recommendations (see Figure 2). The adequacy components such as wholegrains, fruits, total vegetables, dark green leafy and orange vegetables, and fatty acid ratio were given a maximum score of 10 each if the child met the recommended intakes. A minimum score of zero was given when there was no consumption from these food components or if the fatty acid ratio was less than recommended. Proportional scoring was used to calculate intakes from children who consumed in between these limits^3, 6, 7^.

As the over consumption of animal products and refined grains has been highlighted as a potential cause of obesity in children and adolescents^17^, previous studies assessing diet quality of children have reduced scores for overconsumption of these food components^3, 6^. Likewise, this approach was adopted, and scores were reduced proportionately for any consumption of food items from the total rice, meats and milk and dairy products components above the recommendations. Children get a minimum score of zero if they consumed double the recommended servings. For example, consuming eight or more servings of rice and alternatives would give the child zero points (Supplemental Figure 3).

For moderation components such as high sugar foods, SSBs and saturated fats, reverse proportional scoring was conducted. The maximum score of 10 was given if the child adhered to the recommended servings (see Figure 2). Conversely, the minimum score of zero was given if the child exceeded the recommended servings. Proportional scoring was used to calculate intakes from children who consumed in between these limits.

The diet variety component was scored by the number of key food components consumed. The maximum score of 10 was given if the child consumes at least one serving from five key food components, namely total rice and alternatives, fruits, vegetables, meats, and alternatives and milk and dairy products. Conversely, the minimum score of zero was given if the child consumes less than one serving from all the five key food components.

### Dietary assessment

The children were 5 years of age when their diets were assessed over a one-month period between 2015 and 2016 using a validated quantitative FFQ administered to mothers and caregivers by trained interviewers^18^. Portion sizes consumed were estimated by mothers/caregivers using household measures such as slices, cups, spoons etc. The amount consumed for each food item per day was converted to standard serving sizes using the Singapore dietary recommendations for 5-year olds and the Singapore food composition database^12, 19^.

Single food items from the FFQ were directly classified into the DQI-5 components. For example, eggs would be grouped under the meat and alternatives component. Foods from the composite dishes of the FFQ were broken down and allocated to relevant DQI-5 components based on primary ingredients. For example, consuming a savoury sandwich such as a hotdog with bun would contribute to both rice and alternatives and meat and alternatives components.

### Sociodemographic characteristics

Additional information on sociodemographic characteristics such as mothers’ age, ethnicity (Chinese, Indian and Malay), monthly household income in Singapore dollars (high: ≥$6000, middle: $2000-$5999, low: <$2000) and education level (University and above, post-secondary and primary/secondary) were obtained through an interviewer-administered questionnaire at recruitment, between June 2009 and September 2010. The child’s sex and the identity of primary caregiver such as either parents, family members or domestic helpers/nanny was reported by mothers at year 5 clinic visit. The children were measured without shoes for weight and height, using calibrated scales (SECA 813), while standing height was measured using a stadiometer (SECA 213). BMI was calculated from measured weight in kilograms divided by height in meters squared. Age and sex specific BMI Z-scores were derived using WHO growth references^20^. Overweight and obesity were defined as BMI-for-age >2 SD and ≤3 SD of the median and BMI-for-age >3 standard deviations (SD) of the median respectively^20^.

### Construct validity of DQI-5

The DQI-5 was evaluated using a construct validity approach^7, 8, 21^. The construct validity of the DQI-5 was assessed by examining associations between children’s DQI-5 total scores (in tertiles) and their percentage adherence to dietary recommendations for food components, energy intakes and nutrients. Good construct validity will be demonstrated if children with high DQI-5 scores have reported food and nutrient intakes that conform to Singapore dietary recommendations^11-13^. Conversely, children with low DQI-5 scores will have reported food and nutrient intakes that indicate excess consumption of less healthy foods and low intakes of essential nutrients.

### Statistical analysis

FFQ data were available for 788 children. After exclusion of data from twins and infants born prematurely, FFQ data for 767 children were available for analysis (see Figure 1). The normality of the score’s distribution was tested using the Shapiro-Wilk test. The scores for the individual DQI-5 components were not normally distributed, thus described using median and interquartile ranges (IQR). The DQI-5 total scores were normally distributed, therefore described using mean and SD. They were analysed as categorical and continuous variables. DQI-5 total scores were divided by tertiles and the percentage of children adhering to dietary recommendations^22^ within each tertile was calculated. Average intakes of energy and nutrients (carbohydrates, protein, fat, dietary fibre, calcium, iron, beta carotene and vitamin A) were compared across tertiles using a Kruskal-Wallis test. If the test was significant, a Wilcoxon test using the Bonferroni method was carried out to determine which pairs were significantly different. To examine correlations between child and maternal characteristics and DQI-5 total scores (as continuous variable) of the 5-year-old children, a multivariate linear regression model was applied. All analyses were conducted using RStudio software version 1.2.5033^23^. A significance level of p < 0.05 was set throughout.

## Results

### Study population

The characteristics of the children from this study are presented in Table 1. The study sample was composed of children with mothers of Chinese ethnicity 56.6 %, Malay ethnicity 24.9% and Indian ethnicity 18.4%. Of the children, 51.8% were boys and 48.2% were girls. Most of them had a normal BMI (80.7%), while 10.2% and 6.4% were overweight and obese, respectively. From an interviewer-administered questionnaire at recruitment, about half the proportion of children came from households with middle income (52%). Nearly one third of children had mothers with a high education level of university and above (35.3%). The children’s primary caregivers included parents (64.3% of children), family members (12.8%), domestic helpers/nanny (9.3%) and caregiving sharing responsibilities (13.7%).

### DQI-5 scores

The DQI-5 scores were calculated for 767 children and the possible maximum score that could be achieved was 110 points. The total mean (SD) score obtained by the children was 61.6 (13.2) with a score range of 16.8 to 100. Among the 10-point components assessed by median (interquartile range), the components which received high scores included saturated fats: 10.0 (7.9,10.0); total rice and alternatives: 8.6 (6.5, 10.0), fruits: 8.3 (4.5, 10), milk and dairy products: 8.2 (4.0,10.0) and high sugar foods: 7.9 (5.2, 9.5). Children received moderate scores for meat and alternatives: 6.3 (4.0, 9.0), SSBs: 4.8 (1.0, 7.3) and diet variety: 4.0 (4.0, 6.0). The lowest contribution to the DQI-5 scores were from wholegrains: 1.0 (0.0, 6.2), fatty acid ratio: 1.7 (0.0, 3.6) and the two five-point components: total vegetables: 1.3 (0.5, 2.6) and dark green leafy and orange vegetables: 0.9 (0.2, 1.9). The children mostly overconsumed food items from the following components: total milk and dairy products (24.4%), followed by total rice and alternatives (18%) and meat and alternatives (8%) (data not shown).

### Construct validity of DQI-5

Overall, children in the high tertile DQI-5 total scores have significantly greater adherence to most dietary recommendations except for saturated fats as compared to children in the other tertiles (P<0.001) (see Table 2). No significant differences were found among the tertiles for saturated fats (p=0.09). Children in the middle tertile had lower energy intakes compared to other tertiles (p=0.028), while those in the highest tertile consumed significantly more iron and vitamin A than children in the low and middle tertiles (P<0.001). Children in the high tertile were also more likely to meet the RDA for dietary fibre and beta carotene compared to the children in the low tertile (p<0.001). Notably, the majority of children in the high tertile met the dairy products, fruits and vegetables and whole grains recommendations. However, there were no significant differences for % energy from total fat (p=0.649) and calcium intakes (p=0.547) amongst the children.

### Sociodemographic characteristics with DQI-5 score

As presented in Table 3, results showed that the four main correlates of lower DQI-5 scores were belonging to Malay ethnic group (β [95% Cl]:-8.1,[-10.5, -5.8]), with low household income: <$2000 (β [95% CI]:-3.7, [-7.1, 0.2]), having mothers with an educational below university level: primary and secondary (β [95% CI]:-5.6, [-8.4, -2.8]) and post-secondary (β [95% CI]:-3.9, [-6.4, -1.4]) and the primary caregiver having shared responsibility with another caregiver (β [95% CI]:-3.4, [-6.1, -0.7]).The mothers’ age, child’s sex and BMI were not associated with DQI-5 total scores.

## Discussion

In the present study, a DQI for preschool Asian children to measure the diet quality of 5-year-old children in the Singapore GUSTO cohort was developed. While most children adhered to recommendations for saturated fats and high sugar foods, the intakes of wholegrains, vegetables, unsaturated fats, SSBs and diet variety amongst children did not meet recommended levels. The children were found to mostly overconsume food items from the total rice and alternatives and dairy products components. It was observed that children with higher DQI-5 scores were more likely to meet dietary recommendations and have higher intake of nutrients such as dietary fibre, iron, vitamin A and beta carotene. This indicates that the scoring system was successful in grouping the children by diet quality.

### Diet quality of 5-year-olds in the GUSTO cohort

The overall DQI-5 mean score (61.6 of a possible total score of 110) of 5-year-olds in the cohort suggests that there is still capacity for improving the diets of the Singapore children. The results from this study were generally in agreement with those of American children aged 2 to 5 years of age, whose reported intakes were below the recommendations for vegetables, greens and beans, whole grains and fatty acid ratio^24, 25^. Additionally, they are in line with existing limited studies in Singapore which have reported low consumption of fruits, vegetables, and wholegrains in toddlers and school aged children, emphasizing these consistent key dietary concerns in children’s diets^7, 26^. Interestingly, the children in this study appeared to have a better adherence to the dietary recommendation for fruits than vegetables (8.3 out of 10 points vs 1.3 out of 5 points). However, it has been observed in other populations that fruit intake tended to be optimal until age 5, after which, there is a decreasing trend^6, 24^.

In terms of carbohydrate sources, children in this study were not meeting dietary recommendations for wholegrains. Instead, an overconsumption of food items from the total rice and alternatives food group were observed. This trend is similar to preschool children from other countries such as the US and Australia, which reported higher consumption of refined grains over wholegrains^6, 17, 24^. In China, children were reported to have average intakes at 3.4 times the recommendations for grains^6^, while among Greek preschool children, 18% overconsumed grains ^3^. This is of concern as diets generally low in fruits, vegetables and wholegrains are low in micronutrients and dietary fibre^27^ and has health implications such as increased risk of childhood constipation, being malnourished and developing hypercholesterolemia^27, 28^. Excess consumption of refined grains, together with other foods such as animal meat, have also been associated with obesity in children and adolescents ^29^.

In a similar vein, intakes of SSBs exceeding recommendations amongst preschool children in this study are consistent with findings from other studies ^21, 30, 31^. A previous publication in GUSTO children revealed that children consumed higher volumes of SSBs at age 5 as compared to toddlers aged 18 months and this was associated with higher risk of them being overweight or obese.^32^. In this study, it was observed that the most consumed SSB was chocolate malted beverages (data not shown). This is similar to another study where Malaysian preschool children were reported to have flavoured and malted beverages as the most consumed SSB^33^. Next to a high contribution to sugar intake, these flavoured and malted beverages also explain the tendency for children in this study to overconsume dairy products.

For dietary fat intakes, while a significant proportion of children in the present study achieved high scores for saturated fats, low scores for fatty acid ratios were observed. These could be explained by overall low intakes of unsaturated fats and total fats amongst the children (Supplemental Figure 4 and 5), which may be explained by the under-reporting of total fat in the FFQ^18^ and in part the generally low consumption of foods rich in unsaturated fats such as oily fish and nuts and seeds. The lack of differences in total fats, saturated fats and calcium intakes among the children in different DQI tertiles could be explained by children having little variability in fat and milk/dairy intake across the groups.

Overall, it was observed that children with higher DQI scores were more likely to meet most dietary recommendations and have higher intake of most nutrients, suggesting that the DQI scoring system was successful in grouping the children by diet quality.

### Sociodemographic characteristics with DQI-5 score

Children having lower DQI-5 scores were more likely to have mothers with a lower household income and educational level. This finding is consistent with other mother-offspring studies that assessed diet quality and socio-demographic factors^7, 30^. Mothers with inadequate knowledge on the importance of diet quality, or the means to afford such a diet, may not choose the appropriate foods to meet dietary recommendations^34^. Previous studies have reported differences in diet quality amongst different ethnic groups^8, 21, 24^. It was also noted that children with caregivers of shared responsibilities tended to have lower diet quality. This may be attributed to inadequate health knowledge of the caregivers^35^, varying childcare arrangements among the caregivers^36^ or a lack of co-ordination of caregiving responsibilities among caregivers.

Besides the aforementioned factors, other possible factors not examined in this study may also explain why children are not meeting dietary recommendations. In a recent study exploring barriers to healthy dietary behaviours amongst children aged 9-12 years of age in Singapore^37^, it was found that barriers to children consuming a healthy diet included them valuing tasty foods over nutrition, modelling after parents’ unhealthy dietary behaviours, peer influence on fast food consumption, having easy access to fast food chains and convenience stores selling unhealthy snacks ^37^.

### Strengths and limitations

A cohort representative of the Singapore population was used in this study. Furthermore, the dietary data analysed was collected using a validated food frequency questionnaire capturing usual frequency of dietary intake of the children^18^. This DQI-5 incorporated reduction of scores for overconsumption of certain food components and had additional components such as fatty acid ratio and diet variety to assess intake of unsaturated fats whilst limiting saturated fat intake and examines variety consumed from key food components.

There were several limitations in this study that should be considered. The validation of the FFQ used in this study found weak correlations (below 0.40) for carbohydrate, protein, monounsaturated fat, vitamin A and beta carotene intakes. Therefore, the reported absolute nutrient intakes may not be accurate^18^, although still useful for ranking intakes across groups. To further validate the DQI-5, the scores obtained from using the FFQ could have been compared with scores from another dietary assessment method. Although, the Singapore dietary recommendations for preschool children included moderating of sodium and salt intake, this was not included in the DQI-5 as a component. This is due to the challenge in providing accurate estimates of sodium and salt intakes through the FFQ. Lastly, the DQI-5 was only applied on dietary data from 5-year-olds in Singapore, although the recommendations used to develop DQI-5 are also appropriate for three- to six-year-olds. Future work could be done to test the DQI-5 on these ages.

## Conclusion

The DQI-5 developed has validity to assess diet quality for 5-year-old children in Singapore. It successfully determined food components which were inadequately consumed such as wholegrains, vegetables, unsaturated fats and diet variety and highlighted the issue on overconsumption of food items from the total rice and alternatives and dairy products components. These findings could lead to the development of potential intervention strategies towards children meeting dietary recommendations for this age group. Future research could be conducted to examine the relationship between DQI-5 scores and potential health outcomes, such as risk of obesity and related health conditions.

## Supplementary Material

F3

F4

F5

## Figures and Tables

**Table 1:** Sociodemographic characteristics of the children and their mothers included in this study from the Growing Up in Singapore Towards healthy Outcomes cohort study from 2015-2016 (n = 767).

Child characteristics	Mean ± SD or n (%)
**Sex**	
Female	370 (48.2)
Male	397 (51.8)
**BMI at five years of age (Z-score)** ^a^	
Normal	619 (80.7)
Overweight	78 (10.2)
Obese	49 (6.4)
Underweight	15 (2.0)
Missing data	6 (0.8)
**Primary caregiver**	
Parents	493 (64.3)
Family members	98 (12.8)
Domestic helpers/nanny	71 (9.3)
Shared responsibility	105 (13.7)
**Mother characteristics**	**Mean ± SD or n (%)**
**Age (years)**	30.9 ± 5.1
**Ethnicity**	
Chinese	434 (56.6)
Malay	191 (24.9)
Indian	141 (18.4)
Other	1 (0.1)
**Monthly household income (Singapore dollars)^b^**	
≥$6000	213 (27.8)
$2000-$5999	399 (52)
<$2000	107 (13.9)
Missing data	48 (6.2)
**Education level**	
University and above	271 (35.3)
Post-secondary	263 (34.3)
Primary/Secondary	227 (29.6)
Missing data	6 (0.8)

a BMI-for-age z scores at 5 years of age^20^.

b ≥$6000 (High income; equivalent to US$4353.60)

**Table 2:** Adherence to dietary recommendations and recommended energy and nutrient intakes based on tertiles of DQI-5 total scores of 5 year old children from the Growing Up in Singapore Towards healthy Outcomes cohort from 2015-2016 (mean ± SD)

	Tertiles of DQI-5 total score^a^			
	Low tertile n=256	Middle tertile n=255	High tertile n=256	P value
Range of DQI-5 total scores	16.8 - 56.1	56.2 - 66.7	66.8 - 100	
Mean ± SD of DQI-5 scores	47.4 ± 7.6	61.7 ± 3.0	75.9 ± 6.9	
**Percent adherence to recommended**				
**servings or amounts per day (% Mean ± SD)**				
Total rice and alternatives	70.2 ± 27.2	80.4 ± 23.2	86.4 ± 19.6	<0.001^c^
Wholegrains	10.1 ± 21.6	28.3 ± 36.5	56.5 ± 39.9	<0.001^c^
Total fruits	57.5 ± 34.1	69.5 ± 32.8	85.9 ± 23.1	<0.001^c^
Total vegetables	18.8 ± 21.1	32.9 ± 26.7	54.1 ± 32.6	<0.001^c^
Dark green leafy and orange vegetables	12.7 ± 17.8	23.6 ± 23.7	43.9 ± 32.0	<0.001^c^
Total meat and alternatives	48.4 ± 27.6	62.2 ± 26.6	77.0 ± 23.5	<0.001^c^
Total milk and dairy products	51.2 ± 38.5^***^	73.6 ± 30.7	78.9 ± 30.5	<0.001^c^
Fatty acid ratio	13.0 ± 20.1	22.1 ± 23.7	40.6 ± 32.6	<0.001^c^
Sugar sweetened beverages	30.9 ± 31.2	46.1 ± 32.6	56.5 ± 29.3	<0.001^c^
High sugar foods	54.4 ± 35.4	72.6 ± 26.5	80.1 ± 23.3	<0.001^c^
Saturated fats	83.8 ± 22.8	88.2 ± 17.1	89.8 ± 15.9	0.09 ^b^
Diet variety	38.3 ± 15.9	45.4 ± 16.0	58.4 ± 18.8	<0.001^c^
**Energy and nutrients intakes per day^a^ (Mean ± SD)**				
Total Energy (kcal) [1320-1440 kcal]	1527 ± 646	1393 ± 527^*^	1452 ± 410	0.028 ^c^
Carbohydrates (% energy) [45-65% of total energy]	60.3 ± 6.3	59.8 ± 5.6	58.6 ± 5.3	0.002 ^c^
Protein (% energy) [10-15% of total energy]	12.7 ± 2.2	13.4 ± 2.0	14.1 ± 2.0	<0.001^c^
Fat (% energy) [<30% of total energy]	27.0 ± 5.2	26.8 ± 4.5	27.3 ± 4.4	0.649 ^b^
Fibre RDA (g) [17g]	8.9 ± 5.2	9.4 ± 4.8	11.9 ± 6.2	<0.001^c^
Calcium RDA (mg) [600mg]	748.6 ± 463.2	671.8 ± 305.9	692.8 ± 278.4	0.547 ^b^
Iron RDA (mg) [7mg]	10.6 ± 5.5	10.0 ± 4.7	11.2 ± 4.3^***^	<0.001^c^
Beta carotene RDA (mg) [1.8 mg]	0.6 ± 0.8	1.0 ± 0.9	1.7 ± 1.4	<0.001^c^
Vitamin A RDA (μg) [300μg]	422.7 ± 246.4	421.8 ± 185.9	496.9 ±203.3^***^	<0.001^c^

a Energy and nutrient intakes are from the FFQ^18^. Estimated Average Requirement (EAR) values do not exist for the Singaporean population. Therefore, we use the Recommended dietary allowances (RDA) as a measure of adequate nutrient intake. The RDA for energy and nutrients are in brackets and based on the RDA for Singapore children aged 5 years, except for fibre which was based on US RDA for 4 to 8 year olds^22, 25^.

b Intakes were compared across tertiles using a Kruskal-Wallis test and found insignificant with p value >0.05.

c If the Kruskal-Wallis test was significant with p value<0.05, a Wilcoxon test using the Bonferroni method was carried out to determine which pairs were significantly different.

* p value< 0.05

** p value < 0.01

*** p value <0.001.

The tertile presented with relevant number of asterisks denotes that it is statistically different from the other tertiles. The tertiles with no asterisks but with a significant P value denotes that all pairs of tertiles are significantly different from each other.

**Table 3:** Associations between sociodemographic characteristics and total DQI-5 scores of mothers and 5-year-old children in the Growing Up in Singapore Towards healthy Outcomes cohort from 2015-2016 (n = 767).

Sociodemographic characteristics^a^	Multivariate model	
	**β coefficients (95% CI)**	**P value**
**Child characteristics**		
**Sex**		
Female	Reference	-
Male	0.2 (-1.5, 2.0)	0.801
**BMI at 5 years of (Z-score)^b^**	0.1 (-0.7, 0.8)	0.889
**Primary caregiver**		
Parents	Reference	-
Family members	0.0 (-2.8, 2.8)	0.985
Domestic helpers/nanny	-2.2 (-5.4, 0.9)	0.156
Shared responsibility	-3.4 (-6.1, -0.7)	0.013
**Mother characteristics**		
**Age (years)**	0.0 (-0.2, 0.2)	0.927
**Ethnicity**		
Chinese	Reference	-
Indian	-0.1 (-2.6, 2.4)	0.923
Malay	-8.1 (-10.5, -5.8)	<0.001
**Monthly household income (Singapore dollars)^c^**		
≥$6000	Reference	-
$2000-$5999	-0.9 (-3.4,1.5)	0.442
<$2000	-3.7 (-7.1,-0.2)	0.035
**Education**		
University and above	Reference	-
Post-secondary	-3.9 (-6.4, -1.4)	<0.001
Primary/Secondary	-5.6 (-8.4, -2.8)	<0.001

a Missing value: household income: n=48, education level: n=6 and BMI at 5 years age (Z-score): n=6.

b WHO classification of nutritional status using BMI-for-age z-score at 5 years of age^20^.

c ≥$6000 (High income; equivalent to US$4353.60).

## Research snapshot

### Research Question

What is the diet quality of 5-year-old children from the Growing Up in Singapore Towards healthy Outcomes (GUSTO) cohort in Singapore?

### Key Findings

A Diet Quality Index (DQI-5) was developed to assess diets from 767 children aged 5 from the GUSTO mother-offspring cohort. The mean (SD) DQI-5 score was 61.6 (13.2), out of a total of 110 points. Most children adhered to dietary recommendations for saturated fats and high sugar foods, but consumed inadequate wholegrains, vegetables, unsaturated fats and had limited diet variety. Food items from the rice & alternatives and milk & dairy products components tended to be overconsumed.
