# Supplementary material for: Development and evaluation of a Diet Quality Index for preschool children in an Asian population: the GUSTO cohort
Source: J Acad Nutr Diet. Author manuscript; Available in PMC 2023 Feb 1. (PMC7614080; doi:10.1016/j.jand.2022.06.013)
Supplement: F3 [file EMS149032-supplement-F3.docx]

Supplemental Figure 3. Diet quality index scoring system including reduction of scores for overconsumption of total rice, meat, and dairy products amongst five-year olds in the Growing Up in Singapore Towards Healthy Outcomes cohort from 2015-2016.

| **DQI-5 components** | **Recommended servings/**  **Day^a^** | **Number of servings to get a minimum score** | **Proportional Scoring for under consumption** | **Number of servings to get the maximum score** | **Proportional Scoring for over consumption** | **Number of servings to get the minimum score** |
| --- | --- | --- | --- | --- | --- | --- |
| Total rice and alternatives^b^ | 3-4 | 0 | **0 10** | 3-5 | **10 0** | 8 |
| Wholegrains | 0.6-0.8 | 0 | **0 10** | ≥0.6-0.8 |  |  |
| Total fruits | 1 | 0 | **0 10** | ≥1 |  |  |
| Total vegetables | 1 | 0 | **0 5** | ≥1 |  |  |
| Dark green leafy and orange vegetables | 0.5 | 0 | **0 5** | ≥0.5 |  |  |
| Total meat and alternatives^b^ | 1 | 0 | **0 10** | 1-1.5 | **10 0** | 2.5 |
| Total milk and dairy products^b^ | 1 | 0 | **0 10** | 1-2 | **10 0** | 3 |
| Fatty acid ratio | ≥2 | ≤1 | **0 10** | ≥2 |  |  |
| SSBs | 0 ml | ≥250 ml | **0 10** | 0 ml |  |  |
| High sugar foods | <35g added sugar | ≥35g  added sugar | **0 10** | ≤5g  added sugar |  |  |
| Saturated fats | ≤10% total calories | ≥20%  total calories | **0 10** | ≤10%  total calories |  |  |
| Diet variety | Consume at least 1 serving from each of 5 food components | Consume < 1 serving from  all 5 key food components | **0 10** | Consume at least 1 serving from each of 5 food components |  |  |

^a^ The Singapore dietary recommendations for three- to six-year-old children^11-13^.

^b^ Reduction of scores is applied to these DQI-5 food components for overconsumption.
