# Supplementary material for: Development and evaluation of a Diet Quality Index for preschool children in an Asian population: the GUSTO cohort
Source: J Acad Nutr Diet. Author manuscript; Available in PMC 2023 Feb 1. (PMC7614080; doi:10.1016/j.jand.2022.06.013)

Supplemental Figure 4. Histogram on the distribution of unsaturated fatty acids consumed by five-year-old children from the Growing Up in Singapore Towards healthy Outcomes cohort from 2015-2016.


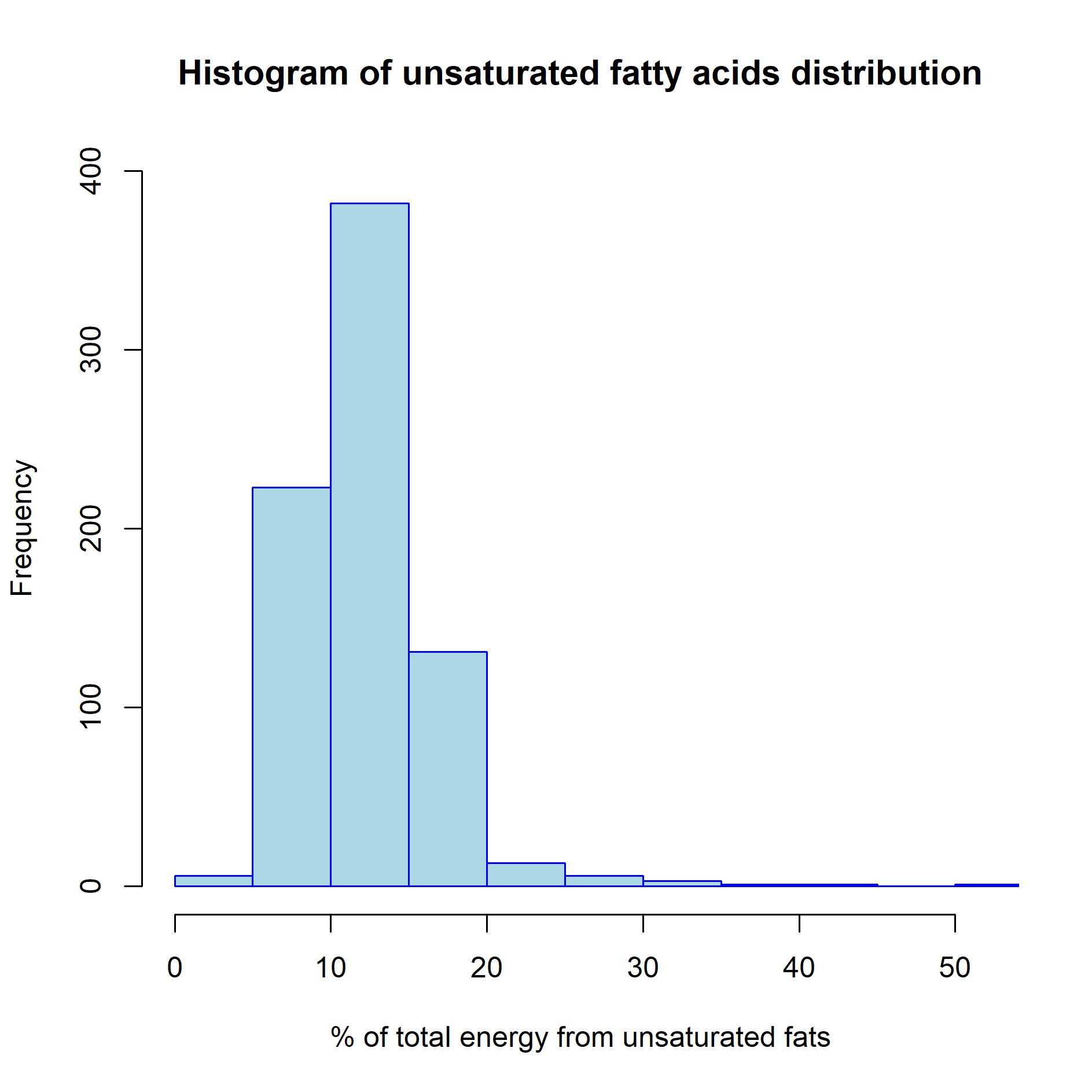

Supplement: F4 [file EMS149032-supplement-F4.docx]
